# What Is a Vitamin? Towards a Contemporary Definition

**DOI:** 10.3390/nu17243890

**Published:** 2025-12-12

**Authors:** Andreas Hahn, Manfred Eggersdorfer, Felix Kerlikowsky

**Affiliations:** 1 Institute of Food and One Health, Leibniz University Hannover, 30167 Hannover, Germany; 2 Society for Applied Vitamin Research E.V. (GVF), 07747 Jena, Germany; 3 Department of Internal Medicine, University Medical Center Groningen, 9713 GZ Groningen, The Netherlands

**Keywords:** vitamins, conditional essentiality, contemporary definition

## Abstract

**Background:** The concept of vitamins has evolved over the past century from compounds preventing classical deficiency diseases to nutrients recognized for supporting long-term health. Despite their central role in science and public health, existing definitions often fail to clearly characterize and distinguish vitamins from other bioactive compounds and do not capture the complexity of their nutritional requirements. **Method:** This article reviews the historical origins and current definitions of vitamins. **Results:** We identify the limitations of existing definitions and present a contemporary, physiologically informed definition as a discussion proposal. Our proposal no longer relies solely on the prevention of classical hypo- or avitaminoses. **Conclusions:** By incorporating the concept of conditional essentiality, this framework also tries to clarify the distinction between classical vitamins and other bioactive substances, reflecting variable dietary requirements under different conditions.

## 1. Historical Background

The scientific investigation of vitamins began with the work of Nicolai Lunin (1853–1937) and Gustav von Bunge (1844–1920), who conducted animal studies demonstrating that life cannot be sustained by the intake of proteins, fats, carbohydrates, salts and water alone. By contrast, administering distinct foods such as milk or egg yolk was sufficient to maintain life, so they postulated that there were “unknown factors in milk” that were essential for life [1]. Dutch physician Christiaan Eijkman (1858–1930) and his student and successor, Gerrit Grijns (1865–1944), were the first to demonstrate that a condition of chicken polyneuritis similar to beriberi was not caused by a bacterial infection, but by the absence of an unknown dietary factor, regardless of macronutrient content [2]. This observation was later emphasized by Frederick Gowland Hopkins (1861–1947), who, in a lecture delivered in London in 1906, established for the first time a connection between nutritional factors and the etiology of diseases such as scurvy and rickets—conditions later recognized as avitaminoses [1]. In 1912, he published results from feeding experiments, thereby illustrating the importance of “accessory factors in normal dietaries” [3]. In 1929, Hopkins and Eijkman were awarded the Nobel Prize in Physiology or Medicine for their contributions to vitamin research. The history of the term “vitamin” dates back to Casimir Funk (1884–1967) in 1912, who wanted to refer to “deficiency substances that are organic bases as ‘vitamins’ [4]. Funk based his hypothesis on the assumption that he had found a substance that was effective against beriberi and that was chemically an amine. That’s why the term “vit-amines” originally implied that these substances were both essential for life (“vital”) and thought to be amines [5]. However, he was wrong in both aspects: the substance was not effective against beriberi, and it was not an amine. Instead, he had discovered nicotinic acid, which was later proved to be active against pellagra. It became clear as vitamins were progressively isolated and chemically characterized that not all vitamins are amines. For example, vitamin C is an oxidized sugar, and vitamin D refers to a collective term of secosteroids [6].

Vitamins were initially classified according to their solubility, following the discovery that milk contains more than one essential nutrient, designated as vitamin A (lipid-soluble and primarily found in cream) and B (water-soluble and found in whey). As research progressed, it became evident that vitamin B comprises multiple chemically and physiologically distinct compounds. Prior to their chemical identification, these compounds were given systematic alphanumeric names: B1, B2, and so on, which are still in use today [7].

Although the term “vitamins” was introduced more than a century ago, there is still no clear and generally accepted definition of what precisely characterizes them or determines which compounds qualify as vitamins. Consequently, several substances once regarded as vitamins, such as salicylic acid, inositol, adenine, and certain fatty acids are no longer classified as such, whereas the status of others, for example, choline, remains controversial [8,9]. These ambiguities raise questions about the clarity and consistency of existing definitions. While understanding of the biochemical and physiological role of vitamins has advanced considerably over the decades, some core elements of their characterization are still based on a traditional understanding of nutrition in general and vitamins in particular. The aim of this work is to further develop the existing criteria and, where necessary, to specify them in order to derive a proposal for discussion for a contemporary definition of the term vitamins.

## 2. Existing Definitions of Vitamins

Over the years, a wide variety of vitamin definitions have been used, whether in specialist journals and textbooks or in publications by authorities and scientific societies. Some of these are listed in Table 1 as examples. It would have gone beyond the scope of this paper to provide even a partial overview of the published definitions. Nevertheless, the literature reveals that there is currently no generally accepted and consistently used definition of the term “vitamins”. Sometimes, even books or chapters on vitamins do not contain any definition at all, possibly because the authors did not even consider the question of what a vitamin is. It is apparent that a wide range of criteria have been and continue to be used to characterize vitamins, although these are by no means found in all definitions. However, only partial reference is made to how the respective criterion is to be understood and what significance it has in classifying substances as vitamins.

## 3. Characteristics of Vitamins

A wide range of criteria has been and continues to be used to characterize vitamins, although these are by no means consistently applied across definitions. The following section aims to outline these criteria, as they appear with varying emphasis in the literature, and to discuss their respective relevance for classifying compounds as vitamins. A useful starting point is the widely acknowledged fact that vitamins are food constituents.

### 3.1. Vitamins Are Food Constituents

It is undisputed that vitamins are substances regularly obtained from food. They are therefore not compounds that occur exclusively in inedible plants or that are originally of synthetic origin. However, this characteristic alone is not sufficient from today’s perspective and has already been modified. Chemically or biotechnologically produced vitamins and derivatives of a vitamin that fulfill its biochemical and physiological functions are also regarded as vitamins [25]. This primarily includes substances used to enrich foods or to formulate food supplements, often for reasons of stability or bioavailability. Examples include retinyl palmitate [26], D-alpha-tocopheryl -succinate [27], inositol hexanicotinate [28], dexpanthenol, cyanocobalamin, L-ascorbyl 6-palmitate, and other derivatives that are likely to be developed in the future.

### 3.2. Vitamins Are Organic Substances

The fact that vitamins are organic compounds is not unique to them, as the macronutrients—proteins, carbohydrates, and fats—are also organic in nature [29]. The reference to their organic character must therefore be understood in the context that vitamins, as micronutrients, differ from minerals, which are inorganic substances typically also required in microgram- to milligram-amounts [30]. This distinguishes both vitamins and minerals from macronutrients.

### 3.3. Vitamins Do Not Constitute a Single Class of Chemical Compounds

Vitamins are chemically diverse molecules belonging to different classes of compounds [29]. Contrary to Casimir Funk’s original assumption, they exhibit considerable structural diversity, resulting in distinct physicochemical properties [31]. A key feature is their division into hydrophilic and lipophilic compounds [29]. Solubility behavior is also associated with fundamental physiological differences, such as lipid-dependent absorption of lipophilic vitamins and renal excretion of hydrophilic ones [32]. For instance, vitamin D is a secosteroid derived from 7-dehydrocholesterol; vitamin A (retinol) is an alcohol; vitamin C is a water-soluble lactone; vitamin B_12_ is a complex cobalt-containing corrinoid; vitamin E consists of lipophilic phenolic compounds; and vitamin K represents lipophilic quinone derivatives [33].

### 3.4. Each Vitamin Encompasses Several Related Compounds

Each vitamin represents a family of structurally related substances, known as vitamers, which include the active forms as well as synthetic derivatives of the corresponding vitamin [33]. These compounds share closely related molecular structures and exhibit the same qualitative biological activity, although their quantitative potency may differ [34]. For example, thiamin (vitamin B1) includes thiamin itself, thiamin monophosphate, thiamin diphosphate, and thiamin triphosphate, with the latter two representing the biologically active forms, as well as synthetic derivatives such as thiamin nitrate [35,36].

Niacin (vitamin B3) occurs as nicotinic acid and nicotinamide, which differ slightly in their physiological properties and potential adverse effects at high doses [37]. Both, as well as nicotinamide mononucleotide (NMN), are converted in the body into the active coenzymes nicotinamide adenine dinucleotide (NAD^+^) and nicotinamide adenine dinucleotide phosphate (NADP^+^), or their reduced forms [38]. Consequently, all of these compounds, including NMN, are considered niacin vitamers.

Pyridoxine (vitamin B6) occurs as pyridoxine, pyridoxal, pyridoxamine, and their respective phosphate esters [37]. The two principal forms of vitamin D are ergocalciferol (D2) and cholecalciferol (D3), with the intermediate calcidiol and the active form calcitriol also included [39]. Vitamin E encompasses eight naturally occurring vitamers—four tocopherols and four tocotrienols (α, β, γ, δ) [40], while vitamin K comprises phylloquinone (K1), a series of menaquinones (K2), and the synthetic derivative menadione (now no longer used in human nutrition for toxicological reasons, however being used in animal nutrition) [41]. The group of folates includes roughly one hundred distinct vitamin-active compounds [37]. These examples illustrate the remarkable structural and functional diversity among vitamins, without implying that the list is exhaustive. For certain vitamins, such as niacin, retinol, or folate, differences in quantitative potency among vitamers are accounted for by expressing their activity in equivalents based on established conversion factors.

### 3.5. Vitamins Are Characterized by Their Specific Effects

Vitamins represent different classes of compounds that are defined not by their chemical structure but by their specific biochemical actions. Consequently, vitamins cannot substitute for one another, even when they share certain structural similarities [28,42].

### 3.6. Provitamins Can Be Converted to Active Vitamins

Several compounds known as provitamins serve as chemically inactive precursors that can be converted by metabolic processes into biologically active vitamins [33]. For example, β-carotene is a provitamin of vitamin A that can be enzymatically cleaved to form active retinol. According to the definitions of the WHO and EFSA, β-carotene and other provitamin A carotenoids are therefore included under the broader term “vitamin A,” as they contribute to its biological activity; for example, α-carotene and β-cryptoxanthin exhibit provitamin A activity at approximately half and one-third of that of β-carotene, respectively [43]. Other substances can likewise be transformed into vitamins in the body and are therefore classified as provitamins. For instance, the degradation of the amino acid tryptophan gives rise, through multiple intermediates, to the coenzymes nicotinamide adenine dinucleotide (NAD^+^) and nicotinamide adenine dinucleotide phosphate (NADP^+^), the active forms of niacin [37]. From this perspective, cholesterol can also be regarded as provitamin D, since it serves as the precursor of 7-dehydrocholesterol in endogenous vitamin D synthesis [44]. Moreover, various synthetic vitamin derivatives act as provitamins, as they are converted into the corresponding active vitamins only after metabolism, for example, pteroylmonoglutamic acid as a precursor of folates [45] or dexpanthenol, which is metabolized to pantothenic acid [46].

### 3.7. Vitamins Fulfill Defined Biochemical Functions

Unlike macronutrients, vitamins do not participate in energy production and are not utilized as structural constituents of tissues or organs. Rather, they exert specific catalytic and regulatory functions within the organism [24]. Their biochemical roles include acting as

- Coenzymes (e.g., B vitamins, vitamin K);
- Ligands for nuclear and membrane-bound receptors (e.g., retinoic acid, calcitriol);
- Acceptors or donors of hydrogen atoms or electrons (e.g., vitamins C and E).

Because of these diverse functions, vitamins are involved in virtually all aspects of intermediary metabolism. Moreover, they play a central role in regulating cell growth and differentiation (vitamins A [47], folate [48], cobalamin [48], and D [49]), constitute integral components of the cellular antioxidant defense system (vitamins C and E [37]), and contribute to maintaining calcium and phosphate homeostasis (vitamin D and vitamin K2) [24,37].

One illustrative example is vitamin C (ascorbic acid), which serves as an essential cofactor for the Fe^2+^- and α-ketoglutarate-dependent dioxygenases prolyl-4-hydroxylase and lysyl-hydroxylase [50]. These enzymes catalyze the post-translational hydroxylation of proline and lysine residues in procollagen, a modification crucial for the formation of thermodynamically stable collagen triple helices. In the absence of vitamin C, this hydroxylation fails because the Fe^2+^ cofactor cannot be regenerated from Fe^3+^. Vitamin C uniquely serves as the reducing agent for prolyl-4-hydroxylase and lysyl-hydroxylase, specifically binding to their active sites and efficiently restoring the Fe^2+^ cofactor without damaging the enzyme or the surrounding cellular environment [50].

This example illustrates how a clearly defined biochemical function—in this case, the maintenance of enzymatic activity through reduction of the iron cofactor—translates into a physiological role by ensuring proper collagen synthesis, thereby explaining the health relevance of the respective vitamin.

### 3.8. Vitamins Are Essential Nutrients

Vitamins are essential, but the concept of nutrient essentiality encompasses two distinct aspects that are often conflated [51,52,53,54]. First, vitamins are biochemically essential: as shown above, this essentiality arises from their specific involvement in defined metabolic processes, making them indispensable for maintaining normal body functions and health.

This must be clearly distinguished from nutritional essentiality, i.e., the requirement for regular intake through food [54]. Vitamins must be supplied in micro- to milligram quantities from the daily diet. To determine whether a substance qualifies as a vitamin, it is essential to consider biochemical and nutritional essentiality separately. In short, a substance must be both biochemically and nutritionally essential to be considered a vitamin in the classical sense.

(A) Biochemical essentiality

A substance can only be classified as a vitamin if it is biochemically essential, meaning that its absence causes defined metabolic disorders and, consequently, functional impairments. Biochemical essentiality is a necessary but not sufficient criterion for vitamin classification, because the metabolism of every organism involves numerous compounds that are essential for specific biochemical reactions; only a subset of these, which must also be obtained from the diet in limited amounts, qualify as vitamins. For this reason, compounds such as phytochemicals and dietary fiber cannot be considered vitamins, because they do not perform biochemical functions that are indispensable for metabolic processes.

Historically, hypo- and avitaminosis were used as practical indicators of a nutrient’s biochemical essentiality [1,23]. The discovery of virtually all vitamins originated from the observation that certain well-known diseases could be traced back to insufficient intake of previously unidentified organic compounds: vitamin C in scurvy, thiamine in beriberi, and cobalamin in pernicious anemia of pregnancy [1]. However, the identification of vitamins historically relied on the prevention or correction of acute deficiency symptoms, implicitly assuming that essentiality manifests through observable clinical signs within a short timeframe. Nutrition science has expanded this concept by recognizing subclinical vitamin deficiency (vitamin insufficiency), in which biochemical or functional impairments occur before overt clinical symptoms develop. Such insufficiencies may still lead to long-term adverse health effects, emphasizing that essentiality cannot be defined solely by the presence or absence of classic deficiency diseases. Modern understanding shows that the traditional approach may overlook substances whose biochemical functions are critical for long-term metabolic integrity and disease prevention.

**Figure 1:**
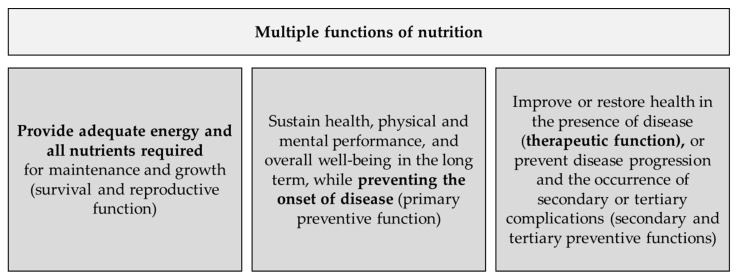
Contemporary scientific perspective of multiple functions of nutrition [24].

From a contemporary perspective, the concept of nutrition must be defined more broadly (Figure 1). Nutrition today serves not only to ensure survival and prevent deficiency symptoms but also to maintain long-term health and prevent diseases [24]. Individualized diets can also play important roles in the management of existing diseases [55].

Against this background, judging biochemical essentiality solely on the presence of hypo- or avitaminosis is insufficient, as this approach relies exclusively on the appearance of specific clinical symptoms as indicators of metabolic disturbance. Folate provides a compelling example: its deficiency impairs cell division and leads to macrocytic hyperchromic anemia—a symptom that develops relatively quickly and thus clearly demonstrates biochemical essentiality [56]. Yet folate also exerts important medium- and long-term functions, such as preventing neural tube defects and regulating DNA methylation [57].

A thought experiment illustrates the limitations of the traditional approach: if folate did not play a role in hematopoiesis and its deficiency therefore caused no anemia, its other essential biochemical functions would probably have remained undiscovered. The effects on neural tube closure become evident only during pregnancy, and disturbances in DNA methylation may contribute to tumor development or other diseases only after many years, often in a multifactorial manner [58]. Under such conditions, folate would not have been recognized as a vitamin, despite its clear biochemical indispensability for long-term health. This example demonstrates that the identification of vitamins based solely on acute deficiency symptoms overlooks substances that are essential for maintaining metabolic integrity and preventing disease over the lifespan.

A substance that has been proposed in this context is vitamin A5 (9-cis-13,14-dihydroretinoic acid; 9CDHRA), first identified and characterized in mice by Rühl and colleagues [59]. 9CDHRA is derived from 9-cis-β,β-carotene and acts primarily as a ligand for retinoid X receptors (RXRs), whereas classical vitamin A compounds act mainly on retinoic acid receptors (RARs) and cannot substitute for RXR-mediated functions [43]. RAR and RXR form heterodimers that repress gene transcription when unliganded and activate transcription when ligand-bound. Some studies suggest partial transcriptional activity if only one receptor is liganded, but maximal co-activator recruitment generally requires ligands at both receptors [60]. A deficiency of the compound called “vitamin A_5_” does not produce the classical manifestations of vitamin A deficiency, such as night blindness, xerophthalmia, or epithelial dysfunction, because it cannot fulfill the canonical functions of vitamin A [43]. Therefore, vitamin A5 cannot currently be considered a vitamer of the vitamin A family, since it is not functionally equivalent to other vitamin A compounds [61]. Experimental evidence in animals suggests potential physiological relevance: in Rbp1-deficient mice, reduced 9CDHRA availability was associated with memory impairments, which could be partially reversed by administration of 9CDHRA. These results indicate a modulatory role in cognitive function, although a direct biochemical link between RXR activation and memory remains unproven [62]. Human data are sparse. While it is conceivable that inadequate availability of provitamin A5/9CDHRA or its dietary precursors might influence neurological health, these effects remain hypothetical. If future research establishes a distinct, essential biochemical function in humans, vitamin A_5_ could be considered a separate, independent vitamin, rather than a member of the classical vitamin A family. Nevertheless, it must be emphasized that any postulated activity or beneficial effect of this compound in humans requires confirmation through direct, evidence-based clinical data.

(B) Nutritional essentiality

As outlined above, the concept of nutritional essentiality refers to the requirement that a given compound must be obtained exogenously through the diet to maintain an adequate nutritional status. This definition is grounded in the inability of the human body to synthesize the substance in sufficient amounts. Conversely, a nutrient classified as nonessential can be synthesized endogenously in quantities adequate to meet physiological needs. This differentiation is not specific to vitamins, but is also used for fatty acids [63], amino acids [64], and minerals [65].

However, the classification is based on a dichotomous principle that appears clear-cut but does not reflect physiological realities [65]. There are numerous substances that cannot be clearly assigned to either group. This is particularly evident in the case of amino acids [64]. Although discussions are still ongoing, a three-way division has now largely become established: essential amino acids, non-essential amino acids, and conditionally essential amino acids. This considers that certain amino acids are only produced in the body, or not produced, under specific conditions [66].

Unlike amino acids, the classification of vitamins is described somewhat differently. It is commonly stated that vitamins cannot be synthesized endogenously, or at least not in sufficient amounts to meet physiological requirements. This means that they have to be regarded as conditionally nutritionally essential nutrients [52]. While an explicit three-tier classification has not been formally incorporated into the official definition of vitamins, the concept has, in effect, been recognized and applied for many years [54].

For example, vitamin D illustrates this principle clearly: the human organism is fundamentally capable of producing vitamin D in the form of cholecalciferol and converting it into its active form, 1,25-dihydroxycholecalciferol—a substance exhibiting properties and effects of a steroid hormone [67]. According to this, vitamin D could be considered non-essential. Nevertheless, it is classified as a vitamin, because it cannot be produced in sufficient quantities under certain conditions. Since endogenous synthesis depends on adequate UV exposure, vitamin D is therefore conditionally nutritionally essential [52,67].

Similarly, niacin can be synthesized endogenously provided that sufficient tryptophan is available; in situations where tryptophan intake is insufficient, niacin becomes conditionally nutritionally essential [68]. Vitamin A may also be classified in this category, since endogenous synthesis from provitamin A carotenoids is only possible if dietary intake of these precursors is adequate [69].

In addition, it is worth considering the potential contribution of the gut microbiome to vitamin status. Certain gut bacteria are capable of synthesizing vitamins [70,71], and evidence indicates that these compounds can be theoretically absorbed in the colon via transport systems in the colonic epithelium [72,73,74,75]. While theoretical models suggest that microbiome-derived vitamins might contribute to daily intake [76], experimental data demonstrating a measurable improvement in host vitamin status are lacking. It also remains unclear whether the vitamins produced are biologically active in humans, and to what extent they are released from bacterial cells for host utilization rather than primarily serving commensal bacteria [76,77].

Importantly, although official definitions do not explicitly recognize a third “conditionally essential” category, this distinction has long been practically applied in nutritional science and has been discussed by several authors [52,54,67].

The fact that some vitamins are conditionally nutritionally essential shows, regardless of whether the term is used or not, that there are a number of substances that cannot be clearly distinguished from vitamins. These include, for example, alpha-lipoic acid, carnitine, choline, glutathione, inositol, taurine, ubichinone (Coenzyme Q10), some of them even called “vitamin-like substances” [34]. Among these biochemically essential substances, i.e., choline, α-lipoic acid (ALA), and carnitine, become nutritionally essential under certain conditions und thus are conditionally nutritionally essential [24]. For example, carnitine can be synthesized in a multistep process from the precursor ε-*N*-trimethyllysine [78]. The synthesis process depends on the availability of several cofactors, including vitamin C, pyridoxal phosphate (PLP), nicotinamide adenine dinucleotide (NAD), and divalent iron [78]. In addition to the dependence of endogenous synthesis on these partly dietary essential factors, genetic defects such as primary and secondary systemic carnitine deficiency, as well as drug interactions (e.g., valproic acid), can impair carnitine metabolism [78]. A vegan diet leads to significantly lower plasma carnitine levels [79]. Carnitine deficiency can occur in two forms. Primary carnitine deficiency is a rare genetic disorder caused by mutations in the OCTN2 transporter, leading to impaired tissue uptake, urinary loss, and systemic depletion, often affecting muscle, heart, and the central nervous system. Secondary carnitine deficiency is more common and arises from excessive urinary loss, metabolic disorders, poor dietary intake, malabsorption, certain medications, or renal disease, typically resulting in milder clinical effects [79]. Consequently, dietary intake of carnitine is vital to prevent deficiency under certain conditions, which places carnitine in a similar category of conditionally nutritionally essential nutrients like vitamin D or niacin.

Choline is another contentious issue. In the United States, it is classified as a vitamin, but in several other countries such as Germany, it is not. Choline has a biochemically essential role in metabolism due to its involvement in the synthesis of phosphatidylcholine and sphingomyelin, two key phospholipids that are vital to the structural integrity of cell membranes [80]. There is considerable discourse regarding the efficiency of endogenous choline synthesis in the human liver, particularly with respect to its conversion to phosphatidylcholine. U.S. institutions, such as the Institute of Medicine and the National Institutes of Health, assert that while the body can synthesize choline, the amount produced is typically insufficient to meet full physiological needs, thereby classifying choline as nutritionally essential. The European Food Safety Authority (EFSA) indicates that choline is considered nutritionally essential in specific circumstances, particularly when endogenous synthesis is inadequate [81]. This can occur, for example, during postmenopausal hormonal changes that reduce the upregulation of the phosphatidylethanolamine *N*-methyltransferase (PEMT) pathway, critical for choline production in the liver. Additionally, in cases of liver dysfunction or conditions like non-alcoholic fatty liver disease (NAFLD), where choline is required for the synthesis of very low-density lipoproteins (VLDL) to transport triglycerides, endogenous production is insufficient, making dietary intake necessary. Thus, choline is conditionally nutritionally essential.

At this point, it is not the intention to discuss in detail whether it is justified to classify these substances as vitamins or not. Rather, the parallel is intended to illustrate that the criterion of nutritional essentiality is not used consistently.

## 4. Towards a Revised Definition of Vitamins

Looking at the criteria described for classifying a substance as a vitamin makes it clear that the definitions used to date are mostly incomplete and, above all, do not correspond to the current state of scientific knowledge. This applies in particular to the question of essentiality, where a strict distinction must be made between biochemical and nutritional essentiality.

It also becomes apparent that the traditional assumption that “vitamins must be supplied with food” is inconsistently applied and, in some cases, cannot be applied at all. Consequently, the distinction between vitamins and, more broadly, vitamin-like substances is not defined in a clear or consistent manner.

**Figure 2:**
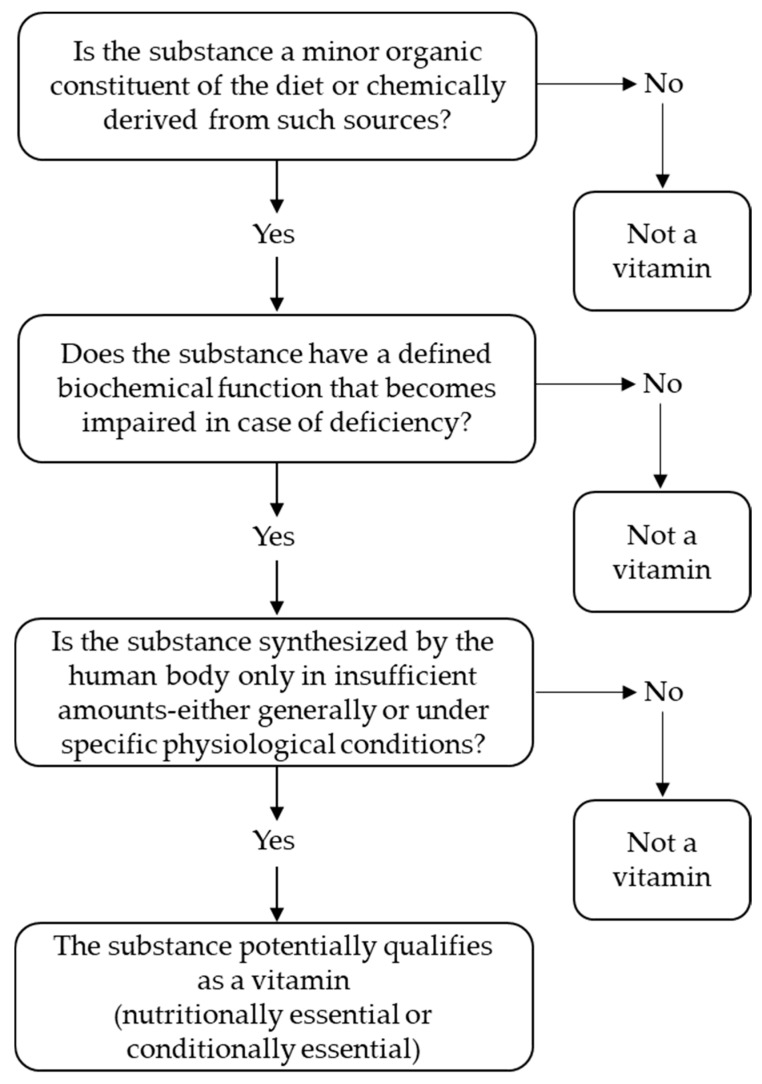
Framework for determining vitamin classification of bioactive compounds.

**Figure 3:**
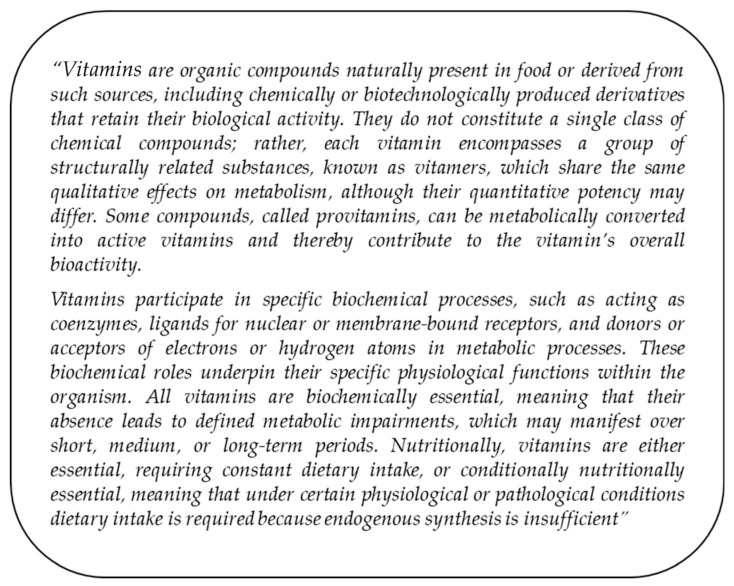
Proposed contemporary definition of vitamins.

In the following, an attempt will be made to illustrate in the form of a checklist **(**Figure 2) how the individual criteria for classification as a vitamin could be evaluated and, at the same time, to define the term ‘vitamin’ in a contemporary way (Figure 3). The authors would like to emphasize that they expressly regard this definition, as well as the entire article, as a proposal for discussion and as an impetus for further debate.

## 5. Conclusions

The traditional approach to defining vitamins exhibit several conceptual limitations, as it relies primarily on the presence of hypo- or avitaminosis. In order to more comprehensively encompass the multiple functions of nutrition, we propose a revised definition of vitamins that integrates the concept of conditional essentiality. This definition is intended to serve as a foundation for scholarly discussion and future refinement.

## Figures and Tables

**Table 1:** Examples of vitamin definitions and main criteria of vitamin definition.

Source	Definition	Organic Compound	Biochemical Essential	Required in Small Amounts	Dietary Intake Necessary	Physiological Function
Encyclopedia Britannica, UK [10]	*“Vitamins are any of several organic substances that are necessary in small quantities for normal health and growth in higher forms of animal life”*	Yes	Implicit (necessary)	Yes	No	Normal health and growth
Encyclopedia Britannica, UK [11]	*“Vitamins are organic compounds found in very small amounts in food and required for normal functioning—indeed, for survival”*	Yes	Implicit (required)	Yes	Yes	Normal functioning, survival
Robert-Koch-Institute (RKI) Germany [12]	*“Vitamins are organic compounds that, with some exceptions (such as vitamin D and niacin), cannot be synthesized by the human body (essential nutrients). Therefore, we depend on obtaining vitamins through our diet. Only milligram or microgram amounts are required from them daily”* [translated by the author]	Yes	Yes	Yes	Yes	/
Society for Applied Vitamin Research (GVF), Germany [13]	*“Vitamins are organic compounds that contribute to the maintenance of physiological functions. Since the human body cannot synthesize vitamins, or cannot produce them in sufficient amounts, it depends on an adequate dietary intake”* [translated by the author]	Yes	No (only contribute)	No	Yes	maintenance of physiological functions
European Food Safety Authority (EFSA) [14]	*“Dietary substance needed in very small amounts to support normal growth and maintenance of health in humans and animals. Most vitamins are “essential” as they are not made within the body”*	No	Implicit	Yes	Yes,	Growth and maintenance of health in humans and animals
World Health Organization (WHO)/Food and Agriculture Organization of the United Nations (FAO) [15]	*“Vitamins are organic substances found in plant and animal foods. Small amounts of vitamins are essential for normal growth and activity of the body”*	Yes	Yes	Yes	No	sustain normal metabolism, growth and physiological functions
U.S. National Library of Medicine (NLM) [16] (U.S.)	*“Vitamins are substances that your body needs to grow and develop normally”*	No	Implicit	No	No	Growth and develop normally
National Cancer Institute (NCI) [17] (U.S.)	*“A nutrient that the body needs in small amounts to function and stay healthy. Sources of vitamins are plant and animal food products and dietary supplements. Some vitamins are made in the human body from food products”*	No	Implicit	Yes	Yes	stay healthy
French Agency for Food, Environmental and Occupational Health & Safety, *Agence nationale de sécurité sanitaire de l’alimentation, de l’environnement et du travail* (ANSES) [18] France	*“Vitamins are substances which have no energy value but which are vital for the body since they are needed for a great many of its physiological processes. Aside from vitamins K and D, the human body cannot synthetise vitamins. Because of this we need to get them though our diet so that our bodies can function correctly”*	No	Implicit	Yes	Yes	great many of its physiological processes
National Health Service (NHS), UK [19]	*“Vitamins and minerals are nutrients your body needs in small amounts to work properly and stay healthy”*	No	Implicit	Yes	No	work properly and stay healthy
American Medical Association (AMA) Vitamin supplementation in disease Prevention [20] U.S.	*“Vitamins are chemically unrelated families of organic compounds that are essential in small amounts for normal metabolism. Because most vitamins cannot be synthesized by humans, they need to be ingested in the diet to maintain health and prevent disease. The exceptions to this are pre-vitamin D3, which is synthesized in the skin following ultraviolet (UV) exposure, and vitamins K2 and B12, which can be synthesized by colonic microbes. These should be distinguished from minerals (such as calcium and iron), some of which are also essential micronutrients”*	Yes	Yes	Yes	Yes	maintain health and prevent disease
National Center for Complementary and Integrative Health, National Institutes of Health (NIH) [21] U.S.	*“Vitamins and minerals are essential substances that our bodies need to function normally”*	No	Yes	No	No	function normally
Quality of Natural Health Products Guide, Health [22] Canada	*“One of a group of naturally occurring organic substances required in small amounts by the body to maintain health; insufficient amounts may cause deficiency diseases”*	Yes	Implicit (required)	Yes	No	maintain health; insufficient amounts may cause deficiency diseases
Nutritional Biochemistry Of The Vitamins [23]	*“The vitamins are a disparate group of compounds; they have little in common* *either chemically or in their metabolic functions. Nutritionally, they form a* *cohesive group of organic compounds that are required in the diet in small* *amounts (micrograms or milligrams per day) for the maintenance of normal* *health and metabolic integrity. They are thus differentiated from the essential* *minerals and trace elements (which are inorganic) and from essential amino* *and fatty acids, which are required in larger amounts”*	Yes	Yes	Yes	Yes	maintenance of normal health and metabolic integrity
Nutrition–Physiological Foundations, Prevention, Therapy [24]	*“Vitamins are organic compounds that are necessary for growth, development, and the maintenance of health, but cannot be produced by the human organism at all or only in insufficient amounts, and therefore must be supplied regularly with the diet in micro- to milligram quantities”* [translated by the author]	Yes	Implicit (necessary)	Yes	Yes	growth, development, and the maintenance of health

## Data Availability

No new data were created or analyzed in this study. Data sharing is not applicable to this article.

## Abbreviations

The following abbreviations are used in this manuscript:

ALA	Alpha-Lipoic Acid
CoA	Coenzyme A
EFSA	European Food Safety Authority
NAD^+^	Nicotinamide Adenine Dinucleotide
NADP^+^	Nicotinamide Adenine Dinucleotide Phosphate
NMN	Nicotinamide Mononucleotide
NHS	National Health Service (UK)
NIH	National Institutes of Health (USA)
NLM	National Library of Medicine (USA)
NCI	National Cancer Institute (USA)
PEMT	Phosphatidylethanolamine *N*-Methyltransferase
PLP	Pyridoxal Phosphate
RAR	Retinoic Acid Receptor
RXR	Retinoid X Receptor
UV	Ultraviolet
VLDL	Very Low-Density Lipoprotein
9CDHRA	9-cis-13,14-dihydroretinoic acid (Vitamin A5)
